# The impacts of knowledge and attitude on behavior of antibiotic use for the common cold among the public and identifying the critical behavioral stage: based on an expanding KAP model

**DOI:** 10.1186/s12889-023-16595-7

**Published:** 2023-08-31

**Authors:** Qianning Wang, Yuanyang Wu, Dan Wang, Xiaoquan Lai, Li Tan, Qian Zhou, Lixia Duan, Rujiao Lin, Xi Wang, Feiyang Zheng, Tiantian Yu, Lu Wang, Si Fan, Yanting Wang, Xinping Zhang, Chenxi Liu

**Affiliations:** 1 School of Medicine and Health Management, Tongji Medical College, Huazhong University of Science and Technology, Wuhan, Hubei China; 2 School of Management, Hubei University of Chinese Medicine, Wuhan, Hubei China; 3 Department of Nosocomial Infection, Tongji Hospital, Tongji Medical College, Huazhong University of Science and Technology, Wuhan, Hubei China; 4 Department of Hospital Infection Management, Tongji Medical College, Wuhan Children’s Hospital (Wuhan Maternal and Child Healthcare Hospital, Huazhong University of Science and Technology, Wuhan, Hubei China

**Keywords:** Knowledge_1_, Attitude_2_, Multi-stage behavior_3_, Perceived threat_4_, Antibiotic use_5_, Common cold_6_, Public_7_, KAP_8_

## Abstract

**Background:**

This study aims to explore the impacts of knowledge and attitude on the behavior of antibiotic use during the treatment of the common cold based on the expanding KAP model, and then identify the critical behavioral stage.

**Methods:**

A cross-sectional study was conducted on 815 public from 21 community health centers (CHCs) in Chongqing, China. Based on the expanding KAP model, a self-administered questionnaire was designed to measure knowledge, attitude, multi-stage behavior, and perceived threat, in which multi-stage behavior was divided into pre-use antibiotic behavior, during-use antibiotic behavior, and post-use antibiotic behavior. A structural equation model was used to examine the model fit and the direct, indirect, mediating effects, and moderating effect of the variables.

**Results:**

The expanding KAP showed good model fit indices with *χ²*/df = 0.537, RMSEA = 0.033, CFI = 0.973, GFI = 0.971, NFI = 0.934, TLI = 0.979. Knowledge had a positive effect on attitude (*β* = 0.503, *p* < 0.05), pre-use antibiotic behavior (*β* = 0.348, *p* < 0.05), during-use antibiotic behavior (*β* = 0.461, *p* < 0.001), and post-use antibiotic behavior (*β* = 0.547, *p* < 0.001). Attitude had a positive effect on during-use antibiotic behavior (*β* = 0.296, *p* < 0.001), and post-use antibiotic behavior (*β* = 0.747, *p* < 0.001). The mediating effect of attitude was positive among knowledge, during-use antibiotic behavior (*β* = 0.149, *p* < 0.05), and post-use antibiotic behavior (*β* = 0.376, *p* < 0.001). Perceived threat also had a positive moderating effect between knowledge and post-use antibiotic behavior (*β* = 0.021, *p* < 0.001).

**Conclusions:**

Knowledge, attitude and perceived threat had different effects on different stages of antibiotic behavior. The critical behavioral stage prioritized the post-use antibiotic behavior and during-use antibiotic behavior over pre-use antibiotic behavior.

## Introduction

Antimicrobial resistance (AR) threatens public health and is a major cause of death globally [1–3]. It was estimated that approximately 7 million deaths are caused by AR worldwide each year, and it would contribute to 10 million deaths by 2050 if effective and immediate actions were not taken [1]. Antibiotic misuse by the public is widely acknowledged as a driver of AR [2], especially during the treatment of the common cold [4, 5]. It was reported that approximately 28% and 45% of the public self-medicated for infections that do not need antibiotics in the United States (U.S.) and Spanish, respectively, such as the common cold and flu [6, 7]. In China, this percentage varies from 7.6 to 82.6%, with the public engaging in unnecessary antibiotic treatment and preventive measures for self-limiting viral infections [8].

Multiple factors contribute to the issue of public antibiotic misuse [9], encompassing elements such as knowledge and attitude toward antibiotic use [10], perceived threat [11], accessibility of antibiotics [12], professionalism of physicians [13, 14], demographics [15], and others. Notably, knowledge and attitude remain primary determinants of antibiotic misuse. In this regard, the Knowledge, Attitude, and Practice (KAP) model serves as a prevalent framework for investigating the behavioral aspect of antibiotic misuse [16]. KAP was first proposed by Hochbaum and used to assess family planning during the middle of the nineteenth century [17, 18]. The KAP model suggests that any behaviors are determined by the person’s attitude and knowledge toward the behavior [19]. However, many studies have found that the current KAP model can not help to accurately judge the relationship between knowledge, attitude, and behavior in the field of antibiotic use [10]. The results of the impacts of knowledge and attitude on behavior in the KAP model were completely different in many studies, in which some studies showed that knowledge and attitude positively affected behavior, while other studies showed that there was no relationship between the three variables [10]. Therefore, a more systematic KAP model is needed to help researchers better observe the behavior of antibiotic use, especially the misuse behavior of the public during the treatment of the common cold.

Research in the field of behavioral psychology provides direction for expanding the KAP model. Behavioral psychologists believe that human behavior is complex unless the behavior is divided into several stages so that the mechanism of the behavior can be observed accurately and the critical behavioral stage can also be identified [20]. A mixed systematic review of the behavior of antibiotic use also advocated treating behavior as a multi-stage process, focusing not only on the current behavior performance but also on the early and later behavior performance [11]. The current behavior performance is likely to be the embodiment of the early behavior performance and the basis of the later behavior performance [11]. Treating behavior as a multi-stage process can help us observe behavior accurately, identify the critical behavioral stage, and prevent further epidemics of antibiotic misuse [11]. Therefore, we expanded the behavior into multi-stage behavior, including pre-use, during-use, and post-use antibiotic behavior based on the basic KAP model.

**Fig. 1:**
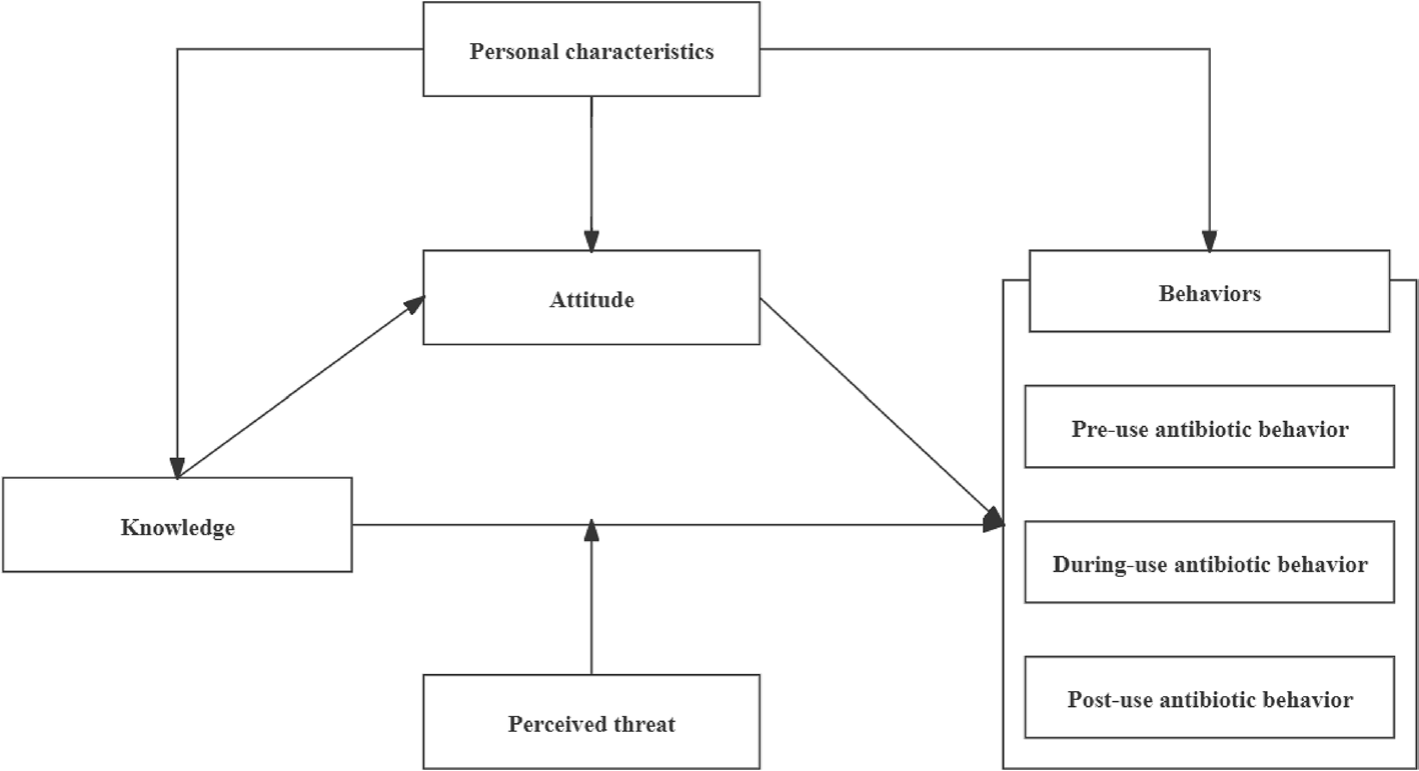
The expanding KAP model

Research in the field of risk management provides another direction for expanding the KAP model. Risk management experts believe that human behavior is easily influenced by the perceived threat from currently unknown but adopted things [21]. Some studies have noted that perceived threat may moderate the behavior of antibiotic misuse [22], but no study has incorporated perceived threat into the KAP model to verify its effectiveness. Further validation of the moderating effect of the perceived threat may help refine the model by explaining the current different results of the impacts of knowledge and attitude on behavior. Therefore, we also incorporated perceived threat into the expanded KAP model. In addition, public demographics may also exert influences on the model [23]. The final expanding KAP model with demographics is shown in Fig. 1.

China is considered the world’s largest consumer of antibiotics for animal and human use [24]. The annual per capita antibiotic use was 138 g, exceeding ten times that of the U.S [25]. The misuse of antibiotics is prevalent in China [26]. A nationwide survey showed that 80% of the patients with colds asked physicians for antibiotics in China, but many of the colds were caused by viruses [27]. There was also an insufficient perception of the threat of antibiotic use in China, 70% of the patients said they took antibiotics by themselves without seeking medical advice [27]. Therefore, in China, it is of great practical significance to understand the impacts of knowledge and attitude on the multi-stage behavior of the public on antibiotic use, as well as the moderating effect of perceived threat in the process.

In order to address the limitations of the current KAP model in validating the behavioral outcomes and better observe the behavior of public antibiotic misuse in China. This study aims to verify the validity of the expanding KAP model, accurately explore the impacts of knowledge and attitude on behavior of antibiotic use during the treatment of the common cold based on the expanding KAP model, and then identify the critical behavioral stage. According to the expanding KAP model, the following hypotheses were examined in this study.

Hypothesis 1: Knowledge is related to attitude.

Hypothesis 2: Attitude is related to multi-stage behavior.

Hypothesis 3: Knowledge is directly related to multi-stage behavior.

Hypothesis 4: Knowledge is related to multi-stage behavior through the mediating effect of attitude.

Hypothesis 5: Perceived threat can moderate the relationship between knowledge and multi-stage behavior.

## Materials and methods

### Settings, sampling and participants

A cross-sectional study using a self-administered questionnaire was conducted in community health centers (CHCs) in Chongqing, China, over the period from July 25 to August 3 in 2022. Chongqing is located in southwest China and has a population of 32.12 million [28]. Its socioeconomic development ranks in the middle (17/31) of all provinces in China [29].

This study covered CHCs in areas with different economic levels, and a stratified cluster random sampling strategy was adopted. Chongqing is economically divided into low-income level areas (annual GDP of fewer than 50 billion yuan), middle-income level areas (annual GDP between 50 billion yuan and 100 billion yuan), and high-income level areas (annual GDP of more than 100 billion yuan) [30]. We randomly selected 1 area from each of the three categories of economic levels as the survey site.

We estimated that at least 21 clusters with a sample size of 35 responses would be required in each CHC using the sample size calculator developed by Dhand and Khatkar (with a level of confidence = 95%, inter-class correlation coefficient = 0.02 and response rate = 0.80) [31]. Therefore, we randomly sampled 7 CHCs at each survey site.

The public aged over 18 years old was approached and invited to participate in the survey. The inclusion criteria of participants were as follows:

1. Experienced symptoms of a common cold (sneezing, runny nose, stuffy nose, cough, hoarseness, body aches, headache, fatigue, etc.) in the past six months [32].
2. Antibiotics taken during the treatment of the common cold in the past six months.
3. Were able to comprehend the survey content.
4. Were able to communicate with the investigators.

### Questionnaire’s measurement

The expanding KAP model required assessing knowledge, attitude, multi-stage behavior, and perceived threat. Besides, respondents’ demographics, such as age and gender, were also collected. The specific measurements are presented as follows.

Knowledge referred to the public’s comprehension of the efficacy and resistance to antibiotics and was measured by 8 items [33, 34], including whether antibiotics can treat colds (viral colds or bacterial colds) and whether antibiotics can develop resistance, etc. The knowledge reliability with Cronbach coefficient alpha value (Cronbach’s alpha) was 0.810. The confirmatory factor analysis (CFA) demonstrated good construct validity (the factor loadings were not less than 0.701). The items were responded with 3-point scale: 1 referred to a correct statement, 2 referred to a false statement, and 3 referred to “unknown”. Respondents were given 1 point for a correct answer and no points for a false answer or for unknown. Higher total correct answer to the eight antibiotic knowledge items indicated a higher knowledge level.

Attitude referred to the public’s preconception of antibiotic use and was measured by 3 items [33, 34], including the effectiveness and side effects of antibiotics, etc. The attitude reliability with Cronbach’s alpha was 0.818, and the factor loadings of CFA were not less than 0.656. The items were responded with 5-point scale: 1 referred to strongly disagree and 5 referred to strongly agree. Higher scores indicated a more correct and higher attitude level.

Multi-stage behavior was measured by 9 items [33], including 3 pre-use antibiotic behavior items, such as information collection, etc., 3 during-use antibiotic behavior items, such as treatment behavior, etc., and 3 post-use antibiotic behavior items, such as future planning, etc. The pre-use antibiotic behavior reliability with Cronbach’s alpha was 0.888, and the factor loadings of CFA were not less than 0.902. The during-use antibiotic behavior reliability with Cronbach’s alpha was 0.905, and the factor loadings of CFA were not less than 0.965. The post-use antibiotic behavior reliability with Cronbach’s alpha was 0.831, and the factor loadings of CFA were not less than 0.862. The item “I treat the cold in a professional way (therapy provided by healthcare professional)” was responded with 7-point scale: 1 to 7 represented treatment ways with gradually increasing professionalism, respectively. Other items were responded with 5-point scale: 1 referred to strongly disagree and 5 referred to strongly agree. Higher scores indicated a greater behavior level.

Perceived threat referred to the public’s judgment of the likelihood of adverse outcomes before using antibiotics and was measured by 3 items [33, 35], including the extent to which respondents perceived the common cold and antibiotic resistance as a threat, etc. The perceived threat reliability with Cronbach’s alpha was 0.837, and the factor loadings of CFA were not less than 0.806. The items were responded with 5-point scale: 1 referred to strongly disagree and 5 referred to strongly agree. Higher scores indicated a higher perceived threat level.

Respondents’ demographics were also collected, including age, gender, facility, education level, occupation, annual household income, whether they had a medical background, and whether they had a chronic disease.

### Data collection and quality control

Each CHC was visited by a pair of trained investigators (one person was added or subtracted according to the size of the CHC). The recruited investigators learned theories and methods of social investigation from their courses and received a one-day intensive training, covering the background of the current survey, detailed interpretation of survey instruments, etc. After a simulation survey test, a total of 10 investigators passed and were recruited in the end.

The investigators approached all of the respondents who met the inclusion criteria in each CHC. The investigators explained the purpose and procedure of the study and obtained written informed consent from each respondent before the respondent was asked to self-complete the questionnaire.

Each investigator was asked to check the quality standards of the questionnaire, including completeness of filling, time appropriateness, and logical rationality of each questionnaire. Based on previous studies, questionnaires with more than 10% missing items were removed [36]. The time required to answer the questionnaire was not less than 8 min (the minimum answer time tested by our research group was 10 min). Our research group set up two items with the same but different expressions in the questionnaire (trap items). If the answers to the two items were inconsistent, we excluded the questionnaire. A token gift (roughly $1.652) was given to the participant upon completion of the survey.

### Statistical analysis

In this study, IBM SPSS Statistics version 25.0 and Amos 28.0 were jointly used to conduct the statistical analyses.

One-way ANOVA or Fisher’s exact test was used to test the difference in the knowledge of the respondents among the three areas of different economic levels. Kruskal-Wallis rank test was used to test the difference in the attitude, multi-stage behavior, and perceived threat of the respondents among the three areas of different economic levels.

A structural equation model (SEM) was applied to explore the relationship among knowledge, attitude, and multi-stage behavior of respondents, as well as the role of perceived threat and demographics in the relationship based on the expanding KAP model. Since the responses of each item were 5 or 7-point scale (ordinal variables), means and variance-adjusted weighted least squares extraction estimation were applied to examine the relations among the study variables.

One-way ANOVA, Fisher’s exact test, and Kruskal-Wallis rank test were considered statistically significant when the *p*-value < 0.05. Goodness of fit indices were applied to evaluate the fit of the SEM: CMIN/DF (*χ²*/df; < 2 excellent), Root mean square error of approximation (RMSEA; < 0.08 acceptable, < 0.05 excellent), Comparative fit index (CFI; > 0.90 acceptable, > 0.95 excellent), Goodness of fit index (GFI; > 0.90 excellent), Normed fit index (NFI; > 0.80 acceptable, > 0.90 excellent), Tucker-Lewis index (TLI; > 0.80 acceptable, > 0.90 excellent) [37].

## Results

### Sample size and demographics of respondents

A total of 908 questionnaires were returned, and 815 were deemed valid after applying quality standards, resulting in an effective response rate of 85.3%.

The 815 respondents had a mean age of 46.244 years (SD = 16.758), and nearly two-thirds of the respondents surveyed were female (60.9%). The respondents were equally distributed among CHCs in low-income level areas (35.2%), middle-income level areas (33.1%), and high-income level areas (31.7%). On average, half of them received junior high school and below education level (56.8%), one-third of them were farmers (29.1%) and had less than 20,000 annual household income (27.5%). Most of them or their family members did not have a medical background (82.3%), and half of them or their family members had chronic disease (50.4%). The demographics of the respondents are presented in Table 1.

**Table 1:** Demographics of respondents (*n* = 815)

Demographic	Mean ± SD / N (%)
**Age (years)**	46.244 ± 16.758
**Gender**	
Male	319 (39.1)
Female	496 (60.9)
**Facility**	
Community health center in low-income level areas	287 (35.2)
Community health center in middle-income level areas	270 (33.1)
Community health center in high-income level areas	258 (31.7)
**Education level**	
Primary school and below	275 (33.7)
Junior high school	188 (23.1)
High school or technical secondary school	114 (14.0)
University or associate and above degree	238 (29.2)
**Occupation**	
Farmer	237 (29.1)
Worker	63 (7.7)
Student	57 (7.0)
Medical worker	31 (3.8)
Teacher	17 (2.1)
Government organ, enterprise or institution worker	81 (10.0)
Self-employed	71 (8.7)
Retiree	63 (7.7)
Unemployed person	111 (13.6)
Other	84 (10.3)
**Annual household income (Chinese RMB ¥ )**	
<20,000	224 (27.5)
20,000 ~	159 (19.5)
40,000 ~	119 (14.6)
60,000~	65 (8.0)
80,000~	73 (8.9)
≥100,000	175 (21.5)
**Have a medical background (Self or family members)**	
Yes	144 (17.7)
No	671 (82.3)
**Have a chronic disease (Self or family members)**	
Yes	411 (50.4)
No	404 (49.6)

### Measurement score of knowledge, attitude, multi-stage behavior and perceived threat of respondents’ antibiotic use for the common cold

As shown in Table 2 (correct items have been indicated in the table), the respondents answered two questions correctly of a total of eight (SD = 1.707). Correct answers were most likely to appear in the item “Antibiotics overuse can lead to antibiotic resistance” (62.8%). Incorrect answers were most likely to appear in the item “Human body can develop resistance to antibiotics” (91.4%). On average, respondents who lived in high-income level areas had higher scores for antibiotic knowledge than other sub-area (*p* < 0.05).

**Table 2:** Measurement score of knowledge of respondents’ antibiotic use for the common cold

Knowledge questions	Number (Percentage) of respondents giving a correct answer				
	Total (*n* = 815)	Community health center in low-income level areas (*n* = 287)	Community health center in middle-income level areas (*n* = 270)	Community health center in high-income level areas (*n* = 258)	*p*-values*
Antibiotics can effectively treat most colds	152 (18.7)	40 (13.9)	55 (20.4)	57 (22.1)	0.031
Antibiotics are anti-inflammatory drugs	126 (15.5)	27 (9.4)	39 (14.4)	60 (23.3)	<0.001
Antibiotics are effective in treating viral cold	102 (12.5)	26 (9.1)	32 (11.9)	44 (17.1)	0.019
Antibiotics are effective in treating bacterial cold^1^	404 (49.6)	136 (47.4)	124 (45.9)	144 (55.8)	0.049
Human body can develop resistance to antibiotics	70 (8.6)	23 (8.0)	27 (10.0)	20 (7.8)	0.614
Bacteria can develop resistance to antibiotics^1^	341 (41.8)	90 (31.4)	111 (41.1)	140 (54.3)	<0.001
Antibiotics overuse can lead to antibiotic resistance^1^	512 (62.8)	159 (55.4)	147 (54.4)	206 (79.9)	<0.001
As long as it has been used for a short time, no antibiotic resistance will occur	195 (23.9)	49 (17.1)	57 (21.1)	89 (34.5)	<0.001
Overall score (Mean ± SD)	2.334 ± 1.707	1.916 ± 1.494	2.193 ± 1.752	2.946 ± 1.714	0.028

Note: *p-values of questions derived from One-way ANOVA (passed by normality test), overall score derived from Fisher’s exact tests. ^1^Statements were considered to be correct. “Total” represented the total number of the respondents who answered the question correctly. The calculation method for the “Overall score” was as follows: the number of questions answered correctly divided by the total number of the questions

As shown in Table 3, the respondents reported the highest attitude score in the item “I fear taking antibiotics regularly will reduce their effectiveness” (Score = 3.407, SD = 1.001). On average, respondents who lived in high-income level areas had higher scores for antibiotic attitude than other sub-area (*p* < 0.001).

**Table 3:** Measurement score of attitude, multi-stage behavior and perceived threat of respondents’ antibiotic use for the common cold

Measurement	Scores (Mean ± SD)				
	Total (*n* = 815)	Community health center in low-income level areas (*n* = 287)	Community health center in middle-income level areas (*n* = 270)	Community health center in high-income level areas (*n* = 258)	*p*-values*
**Attitude**					
Antibiotics can hardly relieve cold symptoms	2.264 ± 0.790	2.171 ± 0.716	2.281 ± 0.837	2.349 ± 0.810	0.028
Antibiotics can hardly reduce the incidence of complications of cold	2.550 ± 0.907	2.376 ± 0.884	2.533 ± 0.890	2.760 ± 0.911	<0.001
I fear taking antibiotics regularly will reduce their effectiveness	3.407 ± 1.001	3.254 ± 1.059	3.300 ± 0.981	3.690 ± 0.894	<0.001
**Multi-stage behavior (pre-use antibiotics)**					
I take cold as a kind of serious disease	2.836 ± 0.895	2.878 ± 1.001	2.800 ± 0.903	2.826 ± 0.752	0.576
I can recognize the severity of cold	3.266 ± 1.158	3.453 ± 1.193	3.100 ± 1.192	3.233 ± 1.051	0.001
I can gather information about how to treat cold	2.444 ± 1.157	2.045 ± 1.081	2.652 ± 1.191	2.671 ± 1.086	<0.001
**Multi-stage behavior (during-use antibiotics)**					
I consult the professional on how to treat cold	2.421 ± 1.459	2.923 ± 1.482	2.352 ± 1.450	1.934 ± 1.254	<0.001
I treat cold in a professional way^1^	5.301 ± 0.686	5.237 ± 0.522	5.311 ± 0.597	5.360 ± 0.899	0.105
I get antibiotics from specialized sources	4.555 ± 0.634	4.732 ± 0.503	4.600 ± 0.600	4.310 ± 0.720	<0.001
**Multi-stage behavior (post-use antibiotics)**					
I find using antibiotics to treat cold have side effects	2.395 ± 1.139	2.084 ± 1.131	2.330 ± 1.166	2.810 ± 0.990	<0.001
I find antibiotics are less effective than other drugs for treating cold	2.560 ± 0.837	2.568 ± 0.767	2.541 ± 0.894	2.570 ± 0.854	0.903
In the future, I will not use antibiotics to treat cold	3.184 ± 0.984	3.045 ± 0.951	3.089 ± 1.070	3.438 ± 0.877	<0.001
**Perceived threat**					
Antibiotic resistance (superbug) is a serious problem in China	3.358 ± 0.835	3.153 ± 0.751	3.352 ± 0.874	3.593 ± 0.824	<0.001
Antibiotic resistance can threaten the health of myself and my family	3.291 ± 0.939	3.230 ± 0.966	3.274 ± 0.932	3.376 ± 0.914	0.182
I was worried that superbugs might harm me and my family	3.494 ± 0.940	3.408 ± 0.967	3.433 ± 0.925	3.655 ± 0.909	0.004

Note: *p-values derived from Kruskal-Wallis rank test. ^1^Responded with 7-point scale: 1 to 7 represented treatment ways with gradually increasing professionalism, respectively; Other items were responded with 5-point scale: 1 referred to strongly disagree and 5 referred to strongly agree. The calculation method for the “Total” was as follows: the respondents’ reported scores divided by the total points of the scale

As for multi-stage behavior, the respondents reported the highest pre-use antibiotic behavior score in the item “I can recognize the severity of cold” (Score = 3.266, SD = 1.158), the respondents reported the highest during-use antibiotic behavior score in the item “I get antibiotics from specialized sources (prescribed by healthcare professional)” (Score = 4.555, SD = 0.634), and the respondents reported the highest post-use antibiotic behavior score in the item “In the future, I will not use antibiotics to treat cold” (Score = 3.184, SD = 0.984). On average, respondents who lived in high-income level areas had higher scores for antibiotic multi-stage behavior in the highest item than other sub-area (*p* < 0.001). The multi-stage behavior of the respondents’ antibiotic use for the common cold is presented in Table 3.

As shown in Table 3, the respondents reported the highest perceived threat score in the item “I was worried that superbugs might harm me and my family” (Score = 3.494, SD = 0.940). Respondents who lived in high-income level areas had higher scores for antibiotic perceived threat than other sub-area in the top two items (*p* < 0.05).

### Relations between knowledge, attitude and multi-stage behavior of respondents’ antibiotic use for the common cold

**Fig. 2:**
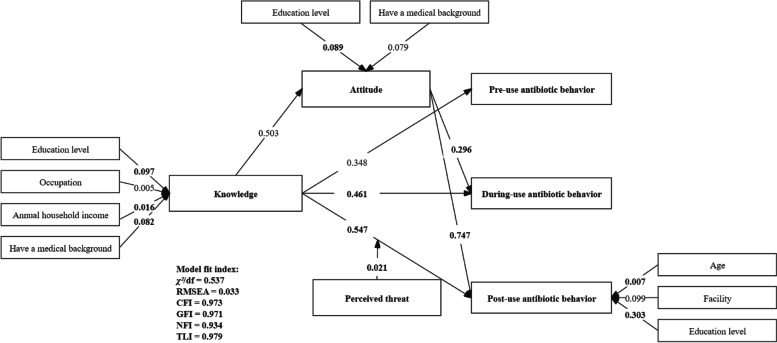
The results of structure equation model on knowledge, attitude, and multi-stage behavior of respondents’ antibiotic use for the common cold, the moderating effect of perceived threat between knowledge and multi-stage behavior, and the effect of demographics on each variable. Note: Only significant pathways were reported with standardized path coefficients. The path coefficients in bold were at the 0.001 level of significance and the others were at the 0.05 level of significance

The SEM based on the expanding KAP showed good model fit indices with *χ²*/df = 0.537 (excellent), RMSEA = 0.033 (excellent), CFI = 0.973 (excellent), GFI = 0.971 (excellent), NFI = 0.934 (excellent), TLI = 0.979 (excellent), and the detailed information is presented in Fig. 2. Knowledge was identified as a significant predictor of attitude and respondents’ pre-use antibiotic behavior, during-use antibiotic behavior, and post-use antibiotic behavior. In addition, attitude was also demonstrated to be a significant predictor of during-use antibiotic behavior and post-use antibiotic behavior.

As shown in Table 4, knowledge had a positive effect on attitude (*β* = 0.503, *p* < 0.05), pre-use antibiotic behavior (*β* = 0.348, *p* < 0.05), during-use antibiotic behavior (*β* = 0.461, *p* < 0.001), and post-use antibiotic behavior (*β* = 0.547, *p* < 0.001). Attitude had a positive effect on during-use antibiotic behavior (*β* = 0.296, *p* < 0.001), and post-use antibiotic behavior (*β* = 0.747, *p* < 0.001). The mediating effect of attitude was positive among knowledge, during-use antibiotic behavior (*β* = 0.149, *p* < 0.05), and post-use antibiotic behavior (*β* = 0.376, *p* < 0.001).

**Table 4:** Direct, indirect, and mediating effects of knowledge, attitude, and multi-stage behavior of respondents’ antibiotic use for the common cold

Path	*β*	SE	*P*	Percentile 95%CI	
				lower	upper
A→B→C1	0.086	0.141	0.082	-0.326	0.012
A→B*	0.503	0.033	0.002	0.265	0.764
B→C1	0.170	0.132	0.181	-0.436	0.059
A→C1	0.348	0.062	0.009	0.162	0.494
A→B→C2	0.149	0.081	0.002	0.352	0.413
B→C2	0.296	0.075	<0.001	0.160	0.449
A→C2	0.461	0.057	<0.001	0.317	0.615
A→B→C3	0.376	0.060	<0.001	0.076	0.165
B→C3	0.747	0.021	<0.001	0.575	0.927
A→C3	0.547	0.031	<0.001	0.442	0.660

Note: A referred to knowledge, B referred to attitude, C1 referred to pre-use antibiotic behavior, C2 referred to during-use antibiotic behavior, and C3 referred to post-use antibiotic behavior; A→B→C1, A→B→C2, and A→B→C3 were mediating effects, A→C1, A→C2, and A→C3 were direct effects, others were indirect effects. *The indirect effects of A→B were the same among A→B→C1, A→B→C2, and A→B→C3

### Moderating effect of perceived threat between knowledge and multi-stage behavior and the effect of demographics on each variable

As shown in Fig. 2, perceived threat had a positive moderating effect between knowledge and post-use antibiotic behavior (*β* = 0.021, *p* < 0.001).

As shown in Fig. 2, the demographics of respondents were also related to their knowledge, attitude, and post-use antibiotic behavior. Respondents with a higher education level had a higher knowledge score (*β* = 0.097, *p* < 0.001), a more correct and higher attitude score (*β* = 0.089, *p* < 0.001), and a higher post-use antibiotic behavior score (*β* = 0.303, *p* < 0.001). Respondents with a medical background had a higher knowledge score (*β* = 0.082, *p* < 0.001) and a more correct and higher attitude score (*β* = 0.079, *p* < 0.05). Respondents with a higher annual household income had a higher knowledge score (*β* = 0.016, *p* < 0.001). Compared with farmers and workers, respondents with a decenter job had a higher knowledge score (*β* = 0.005, *p* < 0.05). In addition, elderly respondents had a higher post-use antibiotic behavior score (*β* = 0.007, *p* < 0.001). Compared with other sub-area, respondents who lived in higher income level areas had a higher post-use antibiotic behavior score (*β* = 0.099, *p* < 0.05).

## Discussion

### Main findings

Our study revealed that the expanding KAP showed good model fit indices. The public held low scores for knowledge, medium scores for attitude and multi-stage behavior, and high scores for perceived threat. Knowledge was a significant predictor of attitude and multi-stage behavior. Attitude was also demonstrated to be a significant predictor of during-use antibiotic behavior and post-use antibiotic behavior. In addition, there were positive mediating effects of attitude between knowledge, during-use antibiotic behavior, and post-use antibiotic behavior. In addition, perceived threat had a positive moderating effect between knowledge and post-use antibiotic behavior, and the demographics of the public were also related to their knowledge, attitude, and post-use antibiotic behavior.

### Discussion on the effectiveness of the expanding KAP model

In previous research on antibiotic knowledge, attitudes, and behaviors, the conventional KAP model was predominantly employed without modifications, although some studies expanded their focus to encompass multiple attitude types [26]. Our study introduced an innovative approach by adopting an expanding KAP model, which integrated behavior as a multi-stage process and included the moderating influence of perceived threat. The model fit indices in our study exhibited excellence, surpassing those of prior research, notably in terms of the RMSEA and TLI indices [38, 39]. The multi-stage behavior holds considerable applicability, yet the moderating effect of perceived threat might manifest instability owing to the impact of cultural variations among nations. Thus, adopting perceived threat as a moderator in Asian regions would be a more favorable approach.

### Discussion on the current status of public antibiotic use and related factors

Congruent with previous studies, the public held a low level of antibiotic knowledge in this study [40–46]. Our study indicated that the public can only correctly answer 25% of the questions on average, most of them mistakenly believed that the human body developed resistance to antibiotics and that antibiotics were effective in treating viral colds. In a global survey conducted by the WHO, three-quarters (76%) of the public believed that antibiotic resistance occurred when the body became resistant to antibiotics [42]. According to a review, an average of 53.9% of the public was unaware that antibiotics were ineffective against viruses [43]. The possible reason is that current public education efforts on antibiotics remain largely superficial and have not yielded the expected results [44–46].

The public exhibited varying attitudes toward different aspects of antibiotic use [47, 48]. Our study demonstrated that the public was concerned about antibiotic resistance, but maintained a neutral attitude toward the efficacy of antibiotics in treating the common cold. Suaifan et al. found that despite 63% of the public being aware of the issue of antibiotic resistance, 65% of them believed that antibiotics were highly effective in treating diseases such as colds and flu [47]. One possible explanation is that the public has already accepted the notion of antibiotic resistance [49], yet it has not fully internalized the fact that antibiotics are ineffective against most cases of the common cold due to their past habit of using antibiotics [50, 51].

The public also exhibited varying behaviors toward different stages of antibiotic use [47, 52–54]. Our study revealed that the public was less proactive in seeking information when pre-use antibiotics, lacked consultation with healthcare professionals when during-use antibiotics, and was neutral in evaluating the treatment outcomes when post-use antibiotics. Eng reported that only 21% of the public searched for information on antibiotics from the internet or other sources [52], Fleming-Dutra found that only 32% of the public sought advice from a healthcare professional [53], and Suaifan evidenced that roughly half of the public still believed that antibiotic therapy was effective in treating the common cold [47]. In contrast to prior studies, we found that the public placed significant importance on preventing the common cold when pre-use antibiotics, obtained medication from appropriate sources and used the medication properly when during-use antibiotics, and exhibited a general willingness to use antibiotics for the treatment of the common cold when post-use antibiotics. One possible explanation is that our study included a higher proportion of middle-aged and elderly people who place greater emphasis on disease prevention [55]. Additionally, the high compliance rate of medication usage among the public in middle and low-income level areas [56], as well as the high rate of correct medication usage among the public in high-income level areas [57], can also partially explain the result.

Consistent with the results of many studies, the public perceived threat to antibiotics was relatively high [58–60]. Our study showed that the majority of the public believed that antibiotic resistance was a serious problem in China and posed a threat to their own and their family’s health. Phagava et al. found that 60.4% of the public considered that antibiotic resistance affected them and their family’s health [58]. The possible reason is that while antibiotic education may still be superficial, it is beginning to have little effect [61, 62]. In the 2016 European Barometer survey, approximately one-third (34%) of the public reported receiving education on antibiotics, leading to some changes in their perceived threat related to antibiotic use [59].

### Discussion on the relationship between public antibiotic use and related factors

Congruent with previous studies, there was a relationship between knowledge, attitude, and behavior [41, 63–67]. Our study showed that knowledge had a positive effect on attitude and multi-stage antibiotic behavior, and attitude also had a positive effect on during-use antibiotic behavior and post-use antibiotic behavior. Karuniawati et al. found a positive correlation between each aspect of knowledge to attitude (0.488), attitude to behavior (0.638), and knowledge to behavior (0.442) [63]. Jairoun et al. reported that the correlation between attitude and pre-use antibiotic behavior was 0.03 [64], while Ajzen et al. reported that the correlations between attitude and behavior, and between attitude and behavioral intention, were 0.35 and 0.67, respectively [65]. The possible reason has been explained by Hochbaum in his work on the KAP model [18], and Kuan et al. also point out that compared to pre-use antibiotic behavior, behavior, and behavioral intention are more likely to be influenced by attitude [68].

In addition, there was a mediating role of attitude [65, 69]. Our study showed that attitude can mediate the relationship between knowledge and behaviors both in during-use antibiotic and post-use antibiotic, and the effect was stronger on post-use antibiotic behavior. Psychologists Ajzen and Fishbein provided evidence for this result, they found the impact of attitude between knowledge and actual behavior was 0.29, while the impact of attitude between knowledge and future behavioral intention was as high as 0.65 [65]. The possible reason is similar to the explanation at the end of the preceding paragraph, which is that post-use behavioral intention is more likely to be mediated by attitude compared to pre-use antibiotic behavior and during-use antibiotic behavior [68].

Innovatively, our study explored the moderating effect of perceived threat within the expanding KAP model. Our study showed that a high public perceived threat increased the impact of knowledge on post-use antibiotic behavior. Sheeran et al. found that perceived threat had a moderating effect of 0.19 on the relationship between knowledge and COVID-19 vaccination behavioral intention, but perceived risk did not have a significant moderating effect on the relationship between knowledge and vaccination actual behavior [70]. One possible reason is that the greater perceived threat increases public concern about using antibiotics, which enhances the impact of knowledge on post-use antibiotic behavior compared to pre-use antibiotic behavior and during-use antibiotic behavior [60, 61].

It has been widely confirmed that individual demographics of the public, such as age, area [15, 71], education level [72], occupation [73], annual family income [74], and medical background [75], have impacts on knowledge, attitude, and behavior [76]. However, the impacts of individual demographics on each variable vary across studies due to differences in background and measurement [77].

Apart from individual factors influencing antibiotic use among the public, the significance of physician training, internet regulations, and pharmacy regulations should also be discussed. A systematic review indicated that enhancing physicians’ rational antibiotic prescribing practices can reduce unrealistic expectations for antibiotics among the public and consequently promote their appropriate utilization [11]. The public used to seek professional or non-professional treatment information through various means, such as the internet. However, the abundance of inaccurate non-professional information necessitates consideration of the effects on the public’s antibiotic cognition and use [78]. Thus, it becomes imperative to prioritize internet regulation to ensure the dissemination of accurate and reliable information. Furthermore, China has enacted regulations to prohibit the non-prescription sale of antibiotics. However, the reality persists where over-the-counter access to antibiotics remains prevalent [79]. Empirical research has demonstrated that antibiotic restriction policies can contribute to a certain extent in curbing antibiotic misuse among the public, though their long-term efficacy might be limited [80]. Hence, diminishing the irrational demand for antibiotics among the public continues to be a pivotal strategy in combating antibiotic misuse.

### Limitation

There were also some limitations in this study. First, it relied on self-report outcomes of the information which depended on the honesty and recall ability of the respondents and thus may be at risk of social desirability bias [81]. Second, the respondents may be more inclined to skim rather than read the questionnaire carefully during busy times and thus measurement deviation may occur (partly driven by the desire to acquire the gift). Third, as this study was conducted in CHCs in one province (Chongqing) of China, the results should be interpreted with caution.

## Conclusion

In this study, SEM was used to verify the validity of the expanding KAP model, accurately explore the impacts of knowledge and attitude on behavior of antibiotic use during the treatment of the common cold based on the expanding KAP model, and then identify the critical behavioral stage.

The main study results revealed that the expanding KAP showed good model fit indices. The public held low scores for knowledge, medium scores for attitude and multi-stage behavior, and high scores for perceived threat. Knowledge had a positive effect on attitude and multi-stage behavior, and attitude also had a positive effect on during-use antibiotic behavior and post-use antibiotic behavior. The mediating effect of attitude was positive between knowledge, during-use antibiotic behavior, and post-use antibiotic behavior. The moderating effect of perceived threat showed a positive relationship between knowledge and post-use antibiotic behavior, and the demographics of the public were also related to their knowledge, attitude, and post-use antibiotic behavior.

This study contributes to the development of the KAP model by expanding it to include knowledge, attitude, multi-stage behavior, and perceived threat. The results help to identify the critical behavioral stage, prioritize the post-use antibiotic behavior and during-use antibiotic behavior over pre-use antibiotic behavior, and suggest the need to focus on public health education and assist the public in establishing a correct perceived threat related to antibiotic use.

## Data Availability

The datasets used and analyzed during the current study are available from the corresponding authors on reasonable request.

## Abbreviations

AR: Antimicrobial Resistance
U.S.: United States
KAP: Knowledge, Attitude, and Practices
CHCs: Community Health Centers
Cronbach Coefficient Alpha Value: Cronbach’s Alpha
CFA: Confirmatory Factor Analysis
SEM: Structural Equation Model
SD: Standard Deviation
SE: Standard Error
95% CI: 95% Confidence Interval
WHO: World Health Organization
